# Uncertainty quantification and sensitivity analysis of an arterial wall mechanics model for evaluation of vascular drug therapies

**DOI:** 10.1007/s10237-017-0944-0

**Published:** 2017-07-28

**Authors:** Maarten H. G. Heusinkveld, Sjeng Quicken, Robert J. Holtackers, Wouter Huberts, Koen D. Reesink, Tammo Delhaas, Bart Spronck

**Affiliations:** 1 Department of Biomedical Engineering, CARIM School for Cardiovascular Diseases, Maastricht University, Universiteitssingel 50, Room 3.358, 6229 ER Maastricht, The Netherlands; 2 Department of Biomedical Sciences, Faculty of Medicine and Health Sciences, Macquarie University, Sydney, NSW Australia; 3 Department of Radiology, CARIM School for Cardiovascular Diseases, Maastricht University, Maastricht, The Netherlands

**Keywords:** Arterial wall mechanics, Constitutive modelling, Uncertainty quantification, Sensitivity analysis, Vascular ultrasound

## Abstract

Quantification of the uncertainty in constitutive model predictions describing arterial wall mechanics is vital towards non-invasive assessment of vascular drug therapies. Therefore, we perform uncertainty quantification to determine uncertainty in mechanical characteristics describing the vessel wall response upon loading. Furthermore, a global variance-based sensitivity analysis is performed to pinpoint measurements that are most rewarding to be measured more precisely. We used previously published carotid diameter–pressure and intima–media thickness (IMT) data (measured in triplicate), and Holzapfel–Gasser–Ogden models. A virtual data set containing 5000 diastolic and systolic diameter–pressure points, and IMT values was generated by adding measurement error to the average of the measured data. The model was fitted to single-exponential curves calculated from the data, obtaining distributions of constitutive parameters and constituent load bearing parameters. Additionally, we (1) simulated vascular drug treatment to assess the relevance of model uncertainty and (2) evaluated how increasing the number of measurement repetitions influences model uncertainty. We found substantial uncertainty in constitutive parameters. Simulating vascular drug treatment predicted a 6% point reduction in collagen load bearing ($L_\mathrm {coll}$), approximately 50% of its uncertainty. Sensitivity analysis indicated that the uncertainty in $L_{\mathrm {coll}}$ was primarily caused by noise in distension and IMT measurements. Spread in $L_{\mathrm {coll}}$ could be decreased by 50% when increasing the number of measurement repetitions from 3 to 10. Model uncertainty, notably that in $L_{\mathrm {coll}}$, could conceal effects of vascular drug therapy. However, this uncertainty could be reduced by increasing the number of measurement repetitions of distension and wall thickness measurements used for model parameterisation.

## Introduction

Arterial stiffening is a major determinant of hypertension and vice versa (Humphrey et al. 2016). Treatment options for arterial stiffening can roughly be divided into two categories: (1) prescribing drugs that lower blood pressure and consequently reverse the arterial structural remodelling that occurs with hypertension, or (2) prescribing drugs that directly affect the arterial wall structure (Townsend et al. 2015). The second category includes cross-link breaking drugs that target the arterial collagen network (Kass et al. 2001; Wolffenbuttel et al. 1998). These types of drugs aim to break down the advanced glycation end products (AGE) that form cross-links between collagen molecules (McNulty et al. 2007; Kass et al. 2001; Brownlee 1995). The desired mechanical effect of such drug therapies is to reverse pressure load bearing from a stiff, collagen-dominated phenotype to a less stiff, elastin-dominated phenotype, resulting in a decrease in material stiffness (O’Rourke and Hashimoto 2007; Wolffenbuttel et al. 1998).

*In vivo* assessment of the performance of vascular drugs has proved to be cumbersome (Engelen et al. 2013). Arterial stiffness is typically quantified by measuring carotid–femoral pulse wave velocity, or by local assessment of arterial distensibility (Kass et al. 2001; Wolffenbuttel et al. 1998). A limitation of these indices is their blood pressure dependence, for which an incremental change in these indices could occur solely from a change in blood pressure (Spronck et al. 2015b). In addition, arterial stiffness measurements as such do not yield insight into the effect of a drug at microstructural level, nor do they resolve whether the load bearing phenotype is collagen- or elastin-dominated.

A potential solution to this problem lies in the use of computer models that simulate stress–strain behaviour of arteries using physical constitutive relations (Holzapfel et al. 2000). In such models, it might be possible to analyse the individual contribution of both elastin and collagen to the overall mechanical response of the vessel wall. *Ex vivo* studies on human carotid arteries, performed in the laboratory, reported good agreement between constitutive model computations and biaxial tensile tests (Sommer and Holzapfel 2012; Sommer et al. 2010). If such models could be parameterised using non-invasive measurements in patients, they could be used to evaluate the mechanics of the vessel wall patient-specifically. Several studies have attempted to use *in vivo* data to parameterise constitutive models of the arterial wall, as reviewed in Spronck et al. (2015a). Typically, diameter, pressure, and intima–media thickness measurements at the carotid artery are used to fit such models (Spronck et al. 2015a). Generally, noise in those measurements will hamper patient-specific evaluation of arterial wall mechanics.

In this study, we aim to (1) assess how measurement uncertainty propagates into uncertainty of mechanical characteristics, estimated using a model of arterial wall mechanics and (2) pinpoint the measurements responsible for the largest spread in mechanical characteristics through sensitivity.

**Fig. 1:**
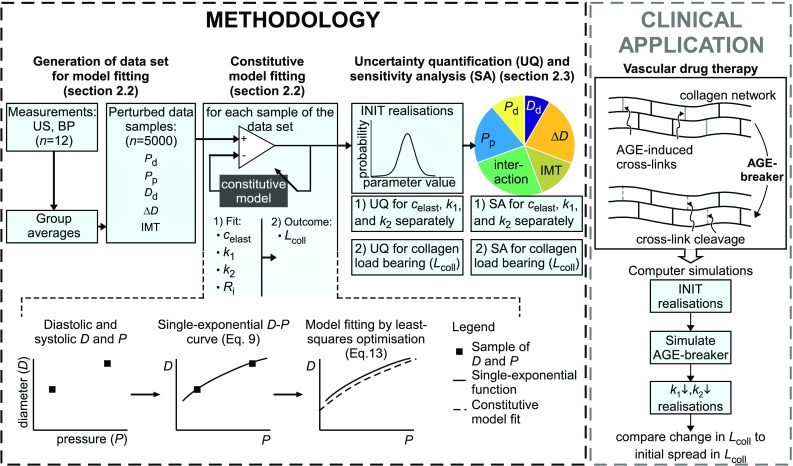
Outline of the current study, involving (1) uncertainty quantification (UQ) and sensitivity analysis (SA) of a constitutive model of arterial wall mechanics (methodology, *left*) and (2) assessment of the relevance of the model output uncertainty when evaluating the effects of vascular drug treatment (clinical application, *right*). *Left* group-averaged measured variables were obtained from a previous study, measuring twelve healthy subjects. The effect of measurement uncertainty on measured variables was included by perturbing group-averaged variables based on their measurement uncertainty. Within the margins of measurement uncertainty, we generated a data set composed of 5000 samples containing measured variables. Here, the measured variables are $D_{\mathrm {d}}$: diastolic diameter, $\Delta D$ distension, $P_{\mathrm {d}}$ and $P_{\mathrm {p}}$ diastolic and pulse pressure, respectively. IMT: intima–media thickness. Holzapfel–Gasser–Ogden models were fitted to single-exponential diameter–pressure curves, determined for each sample of the generated data set. This yielded initial (INIT) realisations of mechanical characteristics, consisting of constitutive parameters and constituent load bearing parameters, that were subsequently used for uncertainty quantification and sensitivity analysis. The constitutive parameters are $c_{\mathrm {elast}}$: elastin stiffness parameter; $k_1$ and $k_2$: collagen stress scaling and stress curve shape parameters, respectively. The parameter $R_{\mathrm {i}}$ indicates the unstressed vessel inner radius and is an auxiliary model parameter. The load bearing parameter $L_{\mathrm {coll}}$ indicates the collagen load bearing as a percentage of the blood pressure (BP) load. *Right* assessing the relevance of uncertainty in load bearing parameters by simulating advanced glycation end product (AGE) breaker treatment using the model. We simulated AGE-breaker treatment by reducing parameters $k_1$ and $k_2$ equally with respect to their INIT best-fit values

Uncertainty quantification and sensitivity analysis are considered indispensable tools to ensure credibility of computer model-based predictions (National Research Council 2012). We explicitly focus on two types of mechanical characteristics: (1) constitutive parameters, describing the mechanical properties of collagen and elastin, and (2) constituent load bearing parameters, describing to which extent the blood pressure load is borne per constituent. The latter parameters are considered outcome parameters, obtained by evaluating the model using the best-fit set of constitutive parameters (Fig. 1, left pane). For these purposes, a large set of virtual pressure (*P*), diameter (*D*), and intima–media thickness (IMT) measurements will be generated by sampling the measurement distributions of previously published *P*, *D*, and IMT measurements in healthy volunteers (Holtackers et al. 2016). For each *D*-*P* sample, we will obtain continuous *D*-*P* curves over the diastolic–systolic pressure range, using the single-exponential function introduced by Hayashi et al. (1980). Holzapfel–Gasser–Ogden constitutive models will be fitted to these data (Holzapfel et al. 2000), and uncertainty quantification will be used to quantify the spread in the estimated mechanical characteristics, given the measurement noise (Fig. 1, left pane). A sensitivity analysis will be performed to determine how noise in the *individual* measured variables propagates into uncertainty in the model-predicted mechanical characteristics. The relevance of the model output uncertainty magnitude, which results from uncertainty in the model parameters, will be assessed by simulating the effect of AGE-breaker treatment on changes in collagen load bearing behaviour in our model (Fig. 1, right pane).

## Methods

### Constitutive model definition

To identify mechanical characteristics of the carotid artery wall, we used the Holzapfel–Gasser–Ogden constitutive model describing passive carotid artery wall mechanics (Holzapfel et al. 2000). This model was chosen on the one hand for its low number of parameters and on the other hand for its feasibility in identifying mechanical characteristics when fitted to clinically obtained distensibility data (Spronck et al. 2015a). Here, we will give a brief description of the model. For more details, we refer to Appendix A1.

**Fig. 2:**
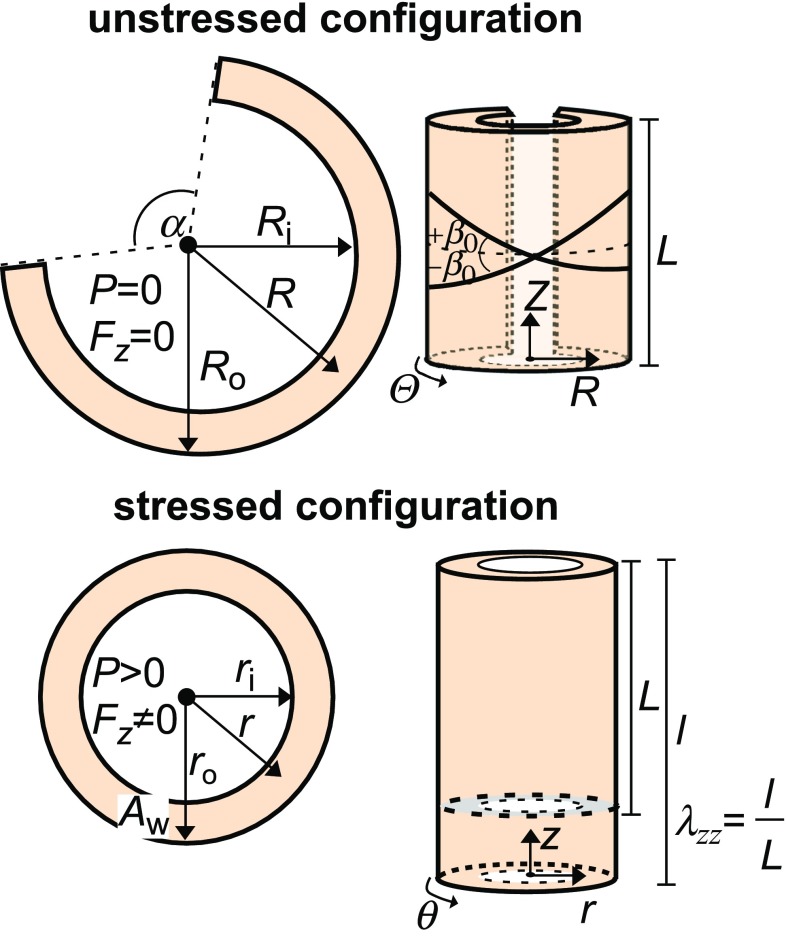
Overview of unstressed (*top*) and stressed (*bottom*) configurations of an artery. *Top* *P*: internal pressure, $F_z$: reduced axial force, *R*: unstressed radius, $R_{\mathrm {i}}$: unstressed inner radius, $R_{\mathrm {o}}$: unstressed outer radius, $\alpha$: opening angle, *L*: unstressed vessel length. In the unstressed configuration, we consider two families of helically orientated collagen fibres, with an angle $\pm \beta _0$ with respect to the circumferential direction. *Bottom* *r*: stressed radius, $r_{\mathrm {i}}$: stressed inner radius, $r_{\mathrm {o}}$: stressed outer radius, $A_\mathrm {w}$: cross-sectional wall area in the stressed configuration, *l*: stressed vessel length, $\lambda _{zz}$: axial pre-stretch

In the model, the carotid artery is considered to be a thick-walled incompressible tubular structure composed of a mixture of two components: (1) elastin, assumed mechanically isotropic, and (2) collagen fibres, assumed mechanically orthotropic. Collagen was modelled by two families of fibres that are helically oriented at an angle of $+\beta _0$ and $-\beta _0$ with respect to the circumferential direction (Fig. 2). Elastin and collagen were assumed to operate in parallel. Furthermore, we assumed that there is only deformation along the principal axes, i.e. no shear. Mechanical properties of the arterial wall components are expressed in terms of strain energy functions.

The strain energy function (SEF) used for elastin ($W_\mathrm {elast}$) is given by

1 $$\begin{aligned} W_{\mathrm {elast}} = c_{\mathrm {elast}}(I_1-3) ~\mathrm {,} \end{aligned}$$

with $I_1$ the first invariant of the Green–Lagrange strain tensor, given by $I_1 = \lambda ^2_{rr} + \lambda ^2_{\theta \theta } + \lambda ^2_{zz}$, with $\lambda$ the stretch in the radial (*r*) direction, circumferential ($\theta$) direction, and axial (*z*) direction, respectively. The parameter $c_{\mathrm {elast}}$ is a constitutive (stiffness) parameter for elastin.

The SEF used for collagen ($W_{\mathrm {coll}}$) is given by

2 $$\begin{aligned} W_{\mathrm {coll}} = \frac{k_1}{k_2} \left[ \exp \left[ k_2\left( \lambda ^2_{\mathrm {fibre}} - 1\right) ^2\right] -1\right] ~{\mathrm {,}} \end{aligned}$$

where $k_1$ and $k_2$ are constitutive parameters and $\lambda _{\mathrm {fibre}}$ denotes fibre stretch, given by $\lambda _{\mathrm {fibre}} =$ $\sqrt{\cos ^2 (\beta _0) \lambda ^2_{\theta \theta } + \sin ^2 (\beta _0) \lambda ^2_{zz}}$. This collagen SEF should only contribute when the fibres are extended (while collagen fibres cannot sustain compression; Holzapfel et al. 2000). The positive luminal arterial pressure used in all our simulations ensures that such condition is always met.

For incompressible materials, the local stress–strain behaviour can be calculated using the derivatives of the SEFs:

3 $$\begin{aligned} \sigma _n = -P_\mathrm {h} + \lambda _n \frac{\partial W_{\mathrm {elast}}}{\partial \lambda _n} + \lambda _n \frac{\partial W_{\mathrm {coll}}}{\partial \lambda _n} ~{\mathrm {,}} \end{aligned}$$

with $\sigma _n$ the Cauchy stress. The subscript *n* is *rr* for the radial direction, $\theta \theta$ for the circumferential direction and *zz* for the axial direction. The pressure $P_{\mathrm {h}}$ is the local hydrostatic pressure within the wall.

In $W_{\mathrm {elast}}$ and $W_{\mathrm {coll}}$, the following parameters thus characterise the constitutive behaviour of elastin and collagen:

- $c_{{\mathrm {elast}}}$: stiffness parameter of elastin, units of Pa.
- $k_1$: stress scaling parameter of collagen, units of Pa.
- $k_2$: collagen stress curve shape parameter, dimensionless.

Elastin acts physiologically as the predominant load bearer for low pressure loads, whereas collagen, which is modelled as an unstressed fibrous material for low pressure, starts bearing load at higher pressures (Holzapfel and Ogden 2010; Watton et al. 2009; O’Rourke and Hashimoto 2007; Shadwick 1999). The fitted parameters should result in a relation that mimics this behaviour. To quantify collagen load bearing, we estimated $L_{\mathrm {coll}}$, being the pressure load borne by collagen as a percentage of the total blood pressure load (see Appendix A1.4).

Based on the literature reports on excised human and animal carotid arteries, we assumed that the artery is subjected to an initial axial pre-stretch ($\lambda _{zz}$, Fig. 2) of 1.20 (Spronck et al. 2015a). The helix angle of the two families of collagen fibres in our model was assumed to be $\pm 35.3^\circ$ in the unstressed configuration (Fig. 2) (Avril et al. 2013). This angle was chosen from an analytical solution that for a thin-walled, fibre-reinforced tube results in a constant reduced axial force ($F_z$) over the cardiac cycle (${\mathrm {d}}F_z/{\mathrm {d}}P = 0$ $\frac{\mathrm {N}}{\mathrm {Pa}}$) (Avril et al. 2013; Humphrey 2002; Takamizawa and Hayashi 1987; Weizsäcker et al. 1983; Van Loon 1976). Here, $F_z$ is defined as the force applied in the axial direction additional to that generated by the pressure on the closed ends of the vessel (Holzapfel and Ogden 2010). Furthermore, in an earlier study fitting a thick-walled two-fibre material model to experimental data from coronary arteries, an optimal fibre angle of $36^\circ$ was found (van der Horst et al. 2012). Cross-sectional wall area ($A_{\mathrm {w}}$, Fig. 2) was calculated from the diastolic outer diameter of the carotid ($D_{\mathrm {d}}$) as well as IMT, using the relation given by

4 $$\begin{aligned} A_{\mathrm {w}} = \pi \left( \frac{1}{4} D^2_{\mathrm {d}} - \left( \frac{1}{2} D_{\mathrm {d}} - \mathrm {IMT}\right) ^2 \right) ~{\mathrm {.}} \end{aligned}$$

**Fig. 3:**
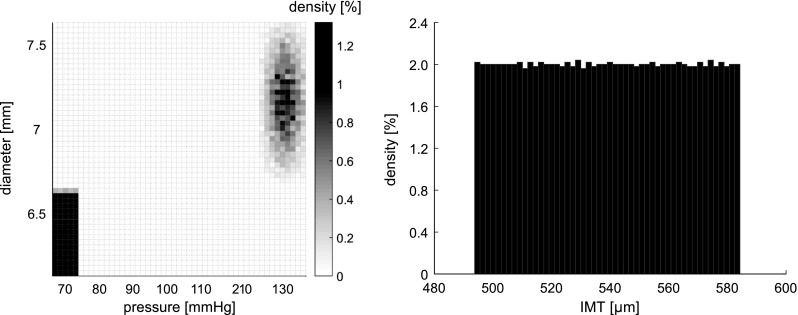
Density plots displaying the distributions of the 5000 computer-generated samples for diastolic and systolic blood pressure and diameter (*left*), as well as intima–media thickness (*right*). Density of diastolic pressure and diameter as well as intima–media thickness samples is high. The greater amount of scattering of systolic diameter and pressure samples arises from the fact that systolic blood pressure is defined as the sum of diastolic and pulse pressure, assumed independent. Similarly systolic diameter is defined as the sum of diastolic diameter and distension, also assumed independent

To map from a cut, stress-free configuration of an artery, to an unloaded intact configuration, to a loaded configuration, we define two additional parameters (Fig. 2, Spronck et al. 2015a; Humphrey 2002; Holzapfel et al. 2002): the opening angle ($\alpha$) and the unstressed inner vessel radius ($R_{\mathrm {i}}$). The value for $\alpha$ was taken from the literature ($100^\circ$, Spronck et al. 2015a). The parameter $R_{\mathrm {i}}$ was fitted using the constitutive model fitting routine (see below).

Residual stresses are accounted for by using the parameters $\lambda _{zz}$, $\alpha$, and $R_{\mathrm {i}}$ to define the stretches in the loaded configuration with respect to the stress-free configuration, as described in Appendix A1. To obtain *D*-*P* and $F_z$-*P* relations, the following boundary value problem was considered. Assuming axisymmetry and neglecting torsion, acceleration forces, body forces and axial extension, the relevant components of the radial momentum balance are given by

5 $$\begin{aligned} \frac{\partial \sigma _{rr}}{\partial r} + \frac{\sigma _{rr} - \sigma _{\theta \theta }}{r} = 0 ~{\mathrm {.}} \end{aligned}$$

Applying the boundary conditions $\sigma _{rr}(r_{\mathrm {i}}) = -P$ and $\sigma _{rr}(r_{\mathrm {o}}) = 0$, with $r_{\mathrm {i}}$ the vessel inner radius and $r_{\mathrm {o}}$ the vessel outer radius, the expression for lumen pressure is given by

6 $$\begin{aligned} P = \int _{r_i}^{r_o} \frac{\sigma _{\theta \theta }-\sigma _{rr}}{r} {\mathrm {d}}r ~{\mathrm {.}} \end{aligned}$$

Reduced axial force is given by

7 $$\begin{aligned} F_z = \pi \int _{r_i}^{r_o} (2\sigma _{zz} - \sigma _{rr} - \sigma _{\theta \theta })r {\mathrm {d}}r ~{\mathrm {.}} \end{aligned}$$

### Parameterisation

#### Clinical measurements

The measurement protocol and data used in the present study are elaborated by Holtackers et al. (2016). Briefly, twelve apparently healthy volunteers ($22\pm 3 \, \mathrm {years}$, 6 males, 6 females) were recruited. The study was approved by the medical ethics committee of Maastricht University Medical Centre (Maastricht, the Netherlands), and written consent was obtained from all participants prior to enrolment.

Ultrasound (US) B-mode recordings were performed at the right common carotid artery (CCA) in the anterolateral plane, and obtained in triplicate. Diastolic blood pressure ($P_{\mathrm {d}}$) and pulse pressure ($P_{{{\mathrm {p}}}}$) were measured three times during the US imaging protocol using an oscillometric device (Omron 705IT; Omron Healthcare Europe, Hoofddorp, the Netherlands).

The US recordings were analysed to determine right CCA cyclic distension (i.e. diastolic–systolic diameter change, $\Delta D$) and diastolic diameter ($D_{\mathrm {d}}$). Because the echo tracking tool used (${}^{\mathrm{RF}}$QAS; Esaote, Maastricht, the Netherlands) utilises the media–adventitia echoes of near and far walls, we assumed the measured diameter signal over time to reflect the CCA *outer* diameter (Spronck et al. 2015a). Furthermore, we obtained IMT at $P_{\mathrm {d}}$ using an automated software tool that reported good agreement with manual IMT methods (Willekes et al. 1999).

#### Generation of data set for constitutive modelling

For the present analysis, we obtained an initial data set consisting of group-averaged values for $P_{\mathrm {d}}$, $P_{{\mathrm {p}}}$, $D_{\mathrm {d}}$, $\Delta D$, and IMT (Fig. 1, left pane). Subsequently, uncertainties in the measured variables were accounted for by generating multiple samples within the uncertainty ranges of each measured metric. We assumed Gaussian distributed uncertainty domains for each measured variable $M_i$ in $\mathbf {M}$, given by

8 $$\begin{aligned} M_i = \bar{M_i} \pm 1.96 \frac{\mathrm {SD}_{\mathrm {intra},M_i}}{\sqrt{N_\mathrm {rep}}} ~{\mathrm {,}} \end{aligned}$$

**Table 1:** Overview of average values, intra-subject SDs, and uncertainty domains per measured variable

Parameter	Unit	Mean	Data of twelve subjects	
			Intra-subject SD	Uncertainty domain
$P_{\mathrm {d}}$	mmHg	72	3.0	[69 ; 75]
$P_{{\mathrm {p}}}$	mmHg	58	3.1	[54 ; 62]
$D_{\mathrm {d}}$	mm	6.37	0.22	[6.12 ; 6.62]
$\Delta D$	mm	0.789	0.035	[0.750 ; 0.829]
IMT	$\upmu \mathrm {m}$	539	40	[494 ; 584]

Intima–media thickness (IMT) was obtained at $P_{\mathrm {d}}$. Intra-subject SD was determined using the method described in Bland and Altman (1996). The uncertainty domains of the measured variables were calculated using their respective mean and intra-subject SD values, see Eq. 8

see also Table 1. Each sample consisted of a vector $\mathbf {M}$ containing the following variables: $\mathbf {M}\,$= [$P_{\mathrm {d}}$, $P_{{\mathrm {p}}}$, $D_{\mathrm {d}}$, $\Delta D$, IMT]. Here, $\bar{M}_i$ represents the average measured value of variable $M_i$ and $\mathrm {SD}_{\mathrm {intra},M_i}$ represents the corresponding intra-subject standard deviation (i.e. indicating measurement error Bland and Altman 1996). In our data set, measurements were performed in triplicate (i.e. $N_\mathrm {rep}=3)$ Holtackers et al. 2016. Samples were generated within the uncertainty domains of the measured variables, using Sobol’s low discrepancy series, implemented in the MATLAB Statistics and Machine Learning Toolbox function sobolset (MATLAB R2015a; The MathWorks, Natick, MA, USA) (Sobol 1967). This sampling method samples the uncertainty domains uniformly and was chosen as a “worst-case scenario.” To ensure adequate convergence of mechanical characteristics distributions, we generated 5000 samples. Systolic blood pressure ($P_\mathrm {s}$) was calculated as $P_\mathrm {s} = P_{\mathrm {d}} + P_{{\mathrm {p}}}$; systolic diameter ($D_\mathrm {s}$) was calculated as $D_\mathrm {s} = D_{\mathrm {d}} + \Delta D$. The distributions of $D_{\mathrm {d}}$, $D_\mathrm {s}$, $P_{\mathrm {d}}$, and $P_\mathrm {s}$ as well as IMT are shown in Fig. 3. The greater amount of scattering of systolic *D* and *P* data points compared to diastolic data points is caused by the fact that systolic blood pressure is defined as the sum of diastolic and pulse pressure. As diastolic and pulse pressure were assumed to be independent, their sum (systolic blood pressure) will have a larger spread than diastolic blood pressure alone. The larger spread in systolic diameter than in diastolic diameter has the same origin, as systolic diameter is defined as the sum of diastolic diameter and distension.

For each sample in the data set, the following single-exponential function was parameterised to obtain continuous *D*-*P* relations:

9 $$\begin{aligned} P(D) = P_{\mathrm {d}} \exp \left[ \gamma \left( \frac{D^2}{D^2_{\mathrm {d}}}-1 \right) \right] ~{\mathrm {.}} \end{aligned}$$

Here, *D* is a continuous variable describing vessel diameter and $\gamma$ is a dimensionless non-linearity factor, which is calculated from systolic and diastolic blood pressures and diameters as described by Hayashi et al. (1980):

10 $$\begin{aligned} \gamma = \frac{ \log \left( \frac{P_\mathrm {s}}{P_{{\mathrm {d}}}}\right) }{\frac{D^2_\mathrm {s}}{D^2_{\mathrm {d}}} -1} ~{\mathrm {.}} \end{aligned}$$

#### Model fitting procedure

The constitutive model was fitted to the single-exponential curve, obtained for each sample $\mathbf {M}$ of the data set. Model fitting was performed by variation of model parameters $c_{{\mathrm {elast}}}$, $k_1$, $k_2$, and $R_{\mathrm {i}}$ (Fig. 1, left pane). Lower and upper bounds of all fitted parameters are given in Table 2.

**Table 2:** Complete overview of lower and upper parameter bounds used for fitting the diameter–pressure data

Parameter	Unit	Lower bound	Upper bound
$c_{\mathrm {elast}}$	kPa	1	400
$k_1$	kPa	0.1 $\times \,10^{-3}$	400
$k_2$	-	0	100
$R_{\mathrm {i}}$	m	0.5 $\times \,10^{-3}$	10 $\times \,10^{-3}$

For each sample in the data set, we assumed the single-exponential curve to be valid within the range $P \in \{P_\mathrm {d,sample} - 15 \, \mathrm {mmHg}, \quad P_\mathrm {s,sample} + 15 \,\mathrm {mmHg}\}$ (Meinders and Hoeks 2004; Hayashi et al. 1980). In Fig. 3 (left), the distribution of $P_\mathrm {d,sample}$ vs. $P_\mathrm {s,sample}$ is displayed.

Fitting was performed using the trust-region reflective algorithm (Moré and Sorensen 1983), implemented in the MATLAB Optimization Toolbox function lsqnonlin, and initiated from 10 random start points in the parameter space using the MATLAB Global Optimization Toolbox function MultiStart. The same 10 start points were used for fitting all samples. Throughout model fitting, we aimed to minimise the sum of squared differences between measured pressure from the single-exponential curve $P_j$ and modelled $P_{\mathrm {mod},j}$:

11 $$\begin{aligned} \epsilon _P = \frac{1}{n_P}\frac{1}{P^2_{{{\mathrm {p}}}}}\sum \limits _{j=1}^{n_P}(P_j-P_{\mathrm {mod},j})^2 ~{\mathrm {,}} \end{aligned}$$

where, for each sample, $n_P$ is the number of fitting points and $P_{{{\mathrm {p}}}}$ is the pulse pressure.

As a physiological constraint, $F_z$ was forced to remain constant at a target value defined $F_{\mathrm {z},\mathrm {target}}$, with varying pressure. This assumption was based on experimental work performed by Van Loon (1976) and Weizsäcker et al. (1983), observing axial force to be nearly insensitive to changes in pressure for arteries inflated at their *in vivo* axial pre-stretch. We enforced this constraint by minimising the following expression:

12 $$\begin{aligned} \epsilon _{F_z} = \frac{1}{n_P}\frac{1}{F^2_{z,\mathrm {target}}}\sum \limits _{j=1}^{n_P}(F_{z,j}-F_{z,\mathrm {target}})^2 ~{\mathrm {,}} \end{aligned}$$

where $F_{z,\mathrm {target}}$ was assumed to be equal to 0.5 N. This value was based on a study by Patel and Fry (1969) in excised, vertically suspended canine arteries that were extended by hanging weights from the bottom end of the artery. For the carotid artery to restore its *in vivo* length, a weight of 54 g was required, corresponding to an $F_z$ of 0.5 N. The two weighted errors $\epsilon _P$ and $\epsilon _{F_z}$ are combined into the weighted total sum of squares ($\epsilon _{\mathrm {T}}$):

13 $$\begin{aligned} \epsilon _{\mathrm {T}} = w_P \epsilon _P + w_{F_z} \epsilon _{F_z} ~{\mathrm {,}} \end{aligned}$$

where $$w_P$$ and $$ w_{F_z}$$ are non-dimensional weighting factors. Here, we chose $$w_P = 10$$ and $$w_{F_z} = 1$$. The $$\epsilon _P$$ term was given a higher importance than the $$\epsilon _{F_z}$$ term, because of the absence of $$F_z$$ measurements in our study and uncertainty in the assumed target value for $$F_z$$. The fitting error describing goodness of fit is expressed as a normalised root mean square error ($$E_{\mathrm {RMS},P}$$ and $$E_{\mathrm {RMS},F_z}$$):

14 $$\begin{aligned} E_{\mathrm {RMS},P}&= 100\% \cdot \sqrt{\epsilon _P}~{\mathrm {,}} \quad ~\mathrm {~and} \end{aligned}$$

15 $$\begin{aligned} E_{\mathrm {RMS},F_z}&= 100\% \cdot \sqrt{\epsilon _{F_z}} ~{\mathrm {.}} \end{aligned}$$

### Simulations and analysis

#### Initial constitutive parameter estimation

The constitutive model was fitted to all 5000 samples of the generated data set using the procedure explained in the previous section. This yielded 5000 initial constitutive model realisations (i.e. termed INIT).

#### Uncertainty quantification and sensitivity analysis

All constitutive model realisations together yield insight in the distribution of the mechanical characteristics that results from the presence of measurement uncertainty. This distribution of mechanical characteristics was therefore used to quantify the uncertainty in constitutive parameters (i.e. the fitted parameters $c_{\mathrm {elast}}$, $k_1$, and $k_2$), as well as collagen load bearing parameters (i.e. the outcome parameter $L_{\mathrm {coll}}$ at various blood pressure levels, Fig. 1, left pane). We used kernel density estimation (KDE) to visualise the distributions of constitutive parameters and load bearing parameters. KDE estimates the probability density function, which in this context implies the probability density of finding a certain value of a constitutive parameter or load bearing value (Silverman 1986). Furthermore, we quantified spread in parameters using the median and the $25\mathrm{th}$ to $75\mathrm{th}$ percentile confidence interval (${\mathrm {PCI}}_{25\rightarrow 75}$). Calculating the ${\mathrm {PCI}}_{25\rightarrow 75}$ comes at hand when assessing spread of skewed distributions.

Sensitivity analysis (SA) was subsequently used to apportion uncertainty in the model-predicted mechanical characteristics to uncertainty in specific measured variables, or their interactions (Huberts et al. 2014). A global variance-based SA was performed using regression-based adaptive generalised polynomial chaos expansion (agPCE), as detailed in Quicken et al. (2016). The agPCE method captures the relation between mechanical characteristics ($X_i$) and measured variables ($M_i$) by means of an adaptively constructed finite polynomial expansion $f_\mathrm {agPCE}$:

16 $$\begin{aligned} X_i = f^{X_i}(\mathbf {M}) \approx f^{X_i}_\mathrm {agPCE}(\mathbf {M}) ~{\mathrm {.}} \end{aligned}$$

Here, $\mathbf {X}$ contains the mechanical characteristics, i.e. $\mathbf {X} = [c_{\mathrm {elast}},k_1,k_2,L_{\mathrm {coll}}]$.

Furthermore, $\mathbf {M} =[M_1,M_2,...,M_{N_\mathrm {vars}}] = [P_{\mathrm {d}}, P_{{\mathrm {p}}}, D_{\mathrm {d}},$ $\Delta D, \mathrm {IMT}]$, and $N_\mathrm {vars}$ is the number of measured variables. Such a polynomial expansion provides a meta-model of the mechanical characteristics estimation method. After constructing the meta-model, the value of the leave-one-out cross-validation coefficient ($Q^2$) was computed. Coefficient $Q^2$, ranging between a value of 0 and 1, is a quality measure of the meta-model, indicating its predictive properties (Sudret 2015). Throughout this study, we assumed $Q^2>0.99$ to indicate an appropriate meta-model.

From the meta-model, the variance of a mechanical characteristic (a measure of its uncertainty) can be computed. The following sensitivity metrics were computed:

- *Main sensitivity indices* The main sensitivity index ($S_{\mathrm {i}}$) of measured variable $M_i$ represents the expected reduction in uncertainty of the mechanical characteristic if $M_i$ were known exactly. Assessment of $S_{\mathrm {i}}$ determines which measured variables are most rewarding to be measured more accurately to reduce model output uncertainty (i.e. parameter prioritisation) (Saltelli et al. 2008).
- *Total sensitivity indices* The total sensitivity index ($S_\mathrm {T}$) of $M_i$ represents the expected uncertainty in the mechanical characteristic that would remain if all other measured variables except $M_i$ were known exactly. Assessment of $S_\mathrm {T}$ determines which measured variables could potentially be fixed within their uncertainty domain (i.e. parameter fixing) (Saltelli et al. 2008).

The contribution of each measured variable to total uncertainty in estimating mechanical characteristics can be illustrated as the segments of a disc (Fig. 1). Total uncertainty may be apportioned to single measured variables but may also arise from interaction between measured variables (Fig. 1). Moreover, significant interaction effects are indicated by large differences between total sensitivity indices and main sensitivity indices (Sudret 2008).

**Fig. 4:**
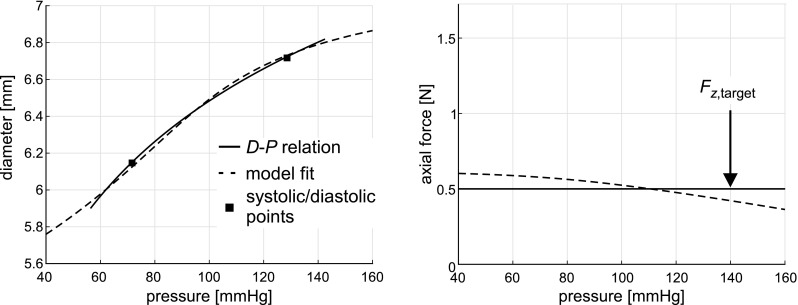
Representative example of the model-predicted diameter–pressure (*D*-*P*) curve and the reduced axial force–pressure ($F_z$-*P*) curve (*dashed lines*) as well as the group-averaged data fitted upon (*solid lines*). The difference between measured and model-predicted pressure was minimised over the pressure fitting range (i.e. $P_{\mathrm {d}} - 15 \, \mathrm {mmHg}$ to $P_\mathrm {s} + 15 \, \mathrm {mmHg}$), whereas reduced axial force was fitted to an assumed target value of 0.5 N ($F_{z,\mathrm {target}}$, respectively). Values of fitted parameters were $c_{\mathrm {elast}} = 40.1 \, \mathrm {kPa}$, $k_1 = 6.9 \, \mathrm {kPa}$ and $k_2 = 8.5$. Fit errors, describing the goodness of fit were $E_{\mathrm {RMS},P} = 0.020\%$ and $E_{\mathrm {RMS},F_z} = 16\%$, respectively

**Fig. 5:**
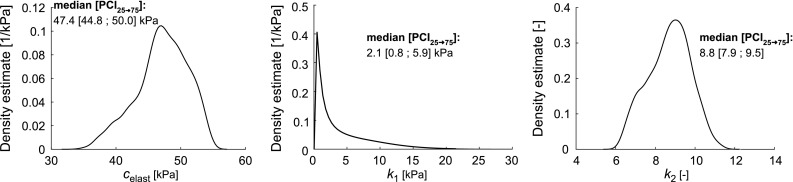
Kernel density estimation (KDE) describing the distributions of fitted constitutive parameters ($c_{\mathrm {elast}}$, $k_1$, and $k_2$). Distributions of parameters were quantified using the median and the $25\mathrm{th}$ to $75\mathrm{th}$ percentile confidence interval (${\mathrm {PCI}}_{25\rightarrow 75}$), respectively

#### Simulating the effect of AGE-breaker vascular drugs

AGE-breaker treatment was simulated using the INIT realisations as take-off points (Fig. 1, right pane). We explicitly assumed that a reduction in cross-link density can be represented in the model by reducing parameters $k_1$ and $k_2$. The rationale of reducing $k_1$ and $k_2$ is based on previous work measuring the stress–strain response of collagen tissue at multiple levels of cross-linking (Kayed et al. 2015; Fratzl 2008). In their work, it was observed that a decrease in cross-link density results in (1) a decrease in fibre stiffness at low amounts of strain and (2) a decrease in the non-linearity of the fibre stress–strain response (Kayed et al. 2015; Fratzl 2008). In our analysis, $k_1$ and $k_2$ were equally reduced by 40% of their initial best-fit value yielding the $k_1\downarrow , k_2\downarrow$ realisations (Fig. 1, right pane). All other constitutive model parameters were assumed to remain unchanged.

## Results

### Representative example of a fitted constitutive model

A representative example of a model-based *D*-*P* and $F_z$-*P* relationship obtained by model fitting a sample within the virtual data set is depicted in Fig. 4. The best-fit constitutive parameters for this representative sample were $c_{\mathrm {elast}}=40.1 \, \mathrm {kPa}$, $k_1 = 6.9 \, \mathrm {kPa}$ and $k_2 = 8.5$. Collagen load bearing ($L_{\mathrm {coll}}$) increased monotonically with blood pressure from 0.6% at diastolic blood pressure ($P_{\mathrm {d}}$), to 10.2% at mean arterial pressure ($P_\mathrm {m}$), up to 25.0% at systolic blood pressure ($P_\mathrm {s}$), respectively. The slope of the plotted curves (${\mathrm {d}}D/{\mathrm {d}}P$) is a measure of vascular compliance. Low vascular compliance corresponds to high vascular stiffness and vice versa. The model fit (dashed lines, Fig. 4) shows sigmoidal *D*-*P* behaviour over a 40 to $160 \, \mathrm {mmHg}$ pressure range, suggesting low compliance for the lower part of pressure range followed by higher compliance in the physiological pressure range and again lower compliance for the upper part of the pressure range. Furthermore, there is some deviation between the model-based $F_z$-*P* curve and the assumed (pressure-independent) target value of 0.5 N (Fig. 4).

**Fig. 6:**
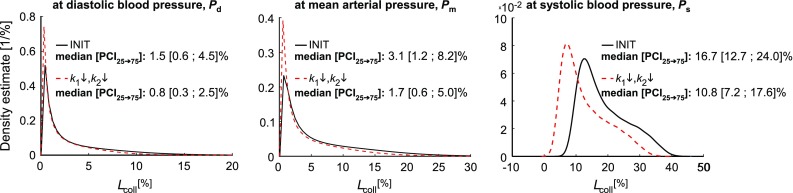
Kernel density estimation (KDE) describing the distribution of collagen load bearing characteristic ($L_{\mathrm {coll}}$). *Black line*: KDEs for initial constitutive model realisations (INIT). *Red dashed line*: KDEs for model with decreased $k_1$ and $k_2$ ($k_1\downarrow ,k_2\downarrow$). KDEs were calculated at three blood pressure levels; diastolic blood pressure ($P_{{\mathrm {d}}}$), mean arterial pressure ($P_{\mathrm {m}}$) and systolic blood pressure ($P_{\mathrm {s}}$). Distributions of parameters were quantified using the median and the 25th to 75th percentile confidence interval (${\mathrm {PCI}}_{25\rightarrow 75}$), respectively

**Table 3:** Main ($S_{\mathrm {i}}$) and total ($S_\mathrm {T}$) sensitivity indices for constitutive parameters $c_{\mathrm {elast}}$, $k_1$ and $k_2$

Measured variable	Symbol	Constitutive parameter					
		$c_{\mathrm {elast}}$		$k_1$		$k_2$	
		$S_{\mathrm {i}}$	$S_\mathrm {T}$	$S_{\mathrm {i}}$	$S_\mathrm {T}$	$S_{\mathrm {i}}$	$S_\mathrm {T}$
Diastolic blood pressure	$P_{\mathrm {d}}$	0.012	0.014	0.00060	0.0011	0.052	0.056
Pulse pressure	$P_{{\mathrm {p}}}$	0.0018	0.021	0.027	0.041	0.10	**0.12**
Diastolic diameter	$D_{\mathrm {d}}$	0.0060	0.036	0.033	0.047	**0.29**	**0.32**
Distension	$\Delta D$	**0.62**	**0.67**	**0.90**	**0.93**	**0.50**	**0.54**
Intima–media thickness	IMT	**0.31**	**0.31**	0.0083	0.015	0.00083	0.00083

Sensitivity indices larger than 0.10 are indicated in bold. The coefficient $Q^2$, indicating the accuracy of the meta-model, was 0.997, 0.998, and 0.998 for $c_{\mathrm {elast}}$, $k_1$, and $k_2$, respectively

**Table 4:** Main ($S_{\mathrm {i}}$) and total ($S_\mathrm {T}$) sensitivity indices for collagen load bearing parameters ($L_{\mathrm {coll}}$) at three blood pressure levels: diastolic, mean, and systolic blood pressure (i.e. $P_{\mathrm {d}}, P_\mathrm {m}$ and $P_\mathrm {s}$, respectively)

Measured variable	Symbol	Load bearing parameter					
		$L_{\mathrm {coll}}$ at $P_{\mathrm {d}}$		$L_{\mathrm {coll}}$ at $P_\mathrm {m}$		$L_{\mathrm {coll}}$ at $P_\mathrm {s}$	
		$S_{\mathrm {i}}$	$S_\mathrm {T}$	$S_{\mathrm {i}}$	$S_\mathrm {T}$	$S_{\mathrm {i}}$	$S_\mathrm {T}$
Diastolic blood pressure	$P_{\mathrm {d}}$	0.00043	0.00098	0.00073	0.0016	0.0019	0.0027
Pulse pressure	$P_{{\mathrm {p}}}$	0.021	0.035	0.023	0.035	0.052	0.054
Diastolic diameter	$D_{\mathrm {d}}$	0.036	0.058	0.041	0.059	0.064	0.067
Distension	$\Delta D$	**0.90**	**0.94**	**0.90**	**0.93**	**0.87**	**0.88**
Intima–media thickness	IMT	0.0028	0.0049	0.0031	0.0046	0.0031	0.0048

Sensitivity indices larger than 0.10 are indicated in bold. The coefficient $Q^2$, indicating the accuracy of the meta-model, was 0.993, 0.995, and 0.999 for diastolic ($P_{\mathrm {d}}$), mean ($P_\mathrm {m}$), and systolic blood pressure ($P_\mathrm {s}$), respectively

### Uncertainty quantification and sensitivity analysis

The distributions of the fitted constitutive parameters are shown in Fig. 5. Best-fit parameter values for elastin stiffness ($c_{\mathrm {elast}}$) were 47.4 [44.8 ; 50.0] (median $[{\mathrm {PCI}}_{25\rightarrow 75}]$). For collagen parameters, best-fit parameter values were 2.1 [0.8 ; 5.9]) kPa for $k_1$, and 8.8 [7.9 ; 9.5]) for $k_2$, respectively.

In Fig. 6, distributions of collagen load bearing parameters ($L_{\mathrm {coll}}$) are given at three blood pressure levels ($P_{\mathrm {d}}$, $P_\mathrm {m}$, and $P_\mathrm {s}$, respectively). Note that $L_{\mathrm {coll}}=0\%$ indicates that blood pressure load is fully borne by elastin, whereas $L_{\mathrm {coll}}=100\%$ indicates that blood pressure load is fully borne by collagen. For the initial constitutive model realisations (INIT), we found $L_{\mathrm {coll}}$ equal to 1.5% at $P_{\mathrm {d}}$, 3.1% at $P_\mathrm {m}$, and 16.7% at $P_\mathrm {s}$ (medians, respectively). As shown in Fig. 6, the $25\mathrm{th}$ to $75\mathrm{th}$ percentile confidence interval for $L_{\mathrm {coll}}$ at $P_{\mathrm {d}}$ as well as $P_\mathrm {s}$ were large ([0.6 ; 4.5]% and [12.7 ; 24.0]%, respectively), indicating large uncertainty in model predictions of constituent load bearing.

A full overview of main and total sensitivity indices is given in Tables 3 and 4. In general, distension and IMT were most rewarding to be measured more reliably. For the constitutive parameters $c_{\mathrm {elast}}$ and $k_1$, this is illustrated by main sensitivity indices ($S_{\mathrm {i}}$) between 0.62 and 0.90 for distension and 0.31 for IMT (Table 3).

**Fig. 7:**
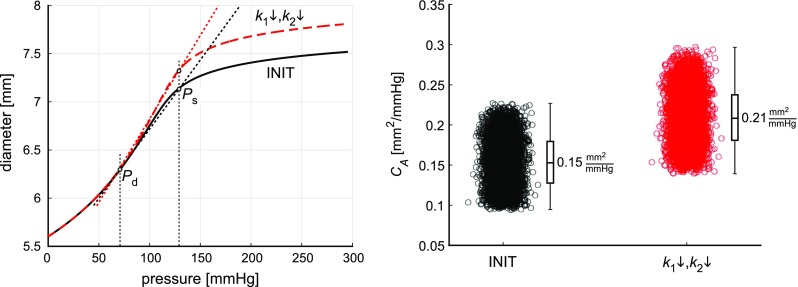
Left pane: Reducing collagen parameters $k_1$ and $k_2$ with 40% (i.e. $k_1\downarrow ,k_2\downarrow$), caused a left-upward shift in the average diameter–pressure (*D*-*P*) relation. Tangent lines (*dotted lines*) from the diastolic *D*-*P* point to the systolic *D*-*P* point indicate area compliance. Right pane: Simulating $k_1\downarrow ,k_2\downarrow$ caused area compliance ($C_A$) to increase from 0.15 $\mathrm {mm}^2/\mathrm {mmHg}$ to 0.21 $\mathrm {mm}^2/\mathrm {mmHg}$, respectively. For each INIT and $k_1\downarrow ,k_2\downarrow$ realisation, area compliance was calculated using $C_A = \left( A_\mathrm {s} - A_{\mathrm {d}}\right) /\left( P_\mathrm {s} - P_{\mathrm {d}}\right)$, with $A_\mathrm {s}=\pi \left( D_{\mathrm {d}}+\Delta D \right) ^2/4$ and $A_{\mathrm {d}}=\pi D_{\mathrm {d}}^2/4$, respectively

For collagen parameter $k_2$, distension and diastolic diameter are most influential (Table 3). Moreover, pulse pressure has some influence, indicated by an $S_{\mathrm {i}}$ of 0.10 (Table 3). For collagen load bearing ($L_{\mathrm {coll}}$), distension was the most important measured variable, indicated by $S_{\mathrm {i}}$ between 0.87 and 0.90 (Table 4). The $S_{\mathrm {i}}$s of the other measured variables were smaller than 0.06, indicating low influence (Table 4). Reducing uncertainty of blood pressure measurements appears of negligible importance in reducing uncertainty in estimating $c_{\mathrm {elast}}$, $k_1$, and $L_{\mathrm {coll}}$, indicated by an $S_{\mathrm {i}}<0.06$ (Tables 3 and 4). Moreover, total sensitivity indices ($S_\mathrm {T}$) for diastolic blood pressure and pulse pressure were smaller than 0.12, suggesting these variables could be fixed in their uncertainty domain. For all measured variables, differences between main and total sensitivity indices were minor, i.e. $S_\mathrm {T} - S_{\mathrm {i}}$ was smaller than 0.10 (Tables 3 and 4). This indicates that the contribution of interaction terms between measured variables to the total variance was negligible (Saltelli et al. 2008).

### Model-based assessment of vascular drug therapies

Figure 7 shows the effect of AGE-breaker treatment (simulated by $k_1\downarrow ,k_2\downarrow$) on the model-predicted *D*-*P* curve, as well as on area compliance ($C_A$). Here, the average *D*-*P* curves, originating on the one hand from the initial best-fit constitutive parameters (INIT, black line), and on the other hand following reduction of $k_1$ and $k_2$ ($k_1\downarrow , k_2\downarrow$, red dashed line), are shown. Moreover, $C_A$ is shown in the right pane for all INIT realisations (black circles) and the $k_1\downarrow , k_2\downarrow$ realisations (red circles). Area compliance was calculated using $C_A = (A_\mathrm {s} - A_{\mathrm {d}})/(P_\mathrm {s} - P_{\mathrm {d}})$, with $A_\mathrm {s}=\pi \left( D_{\mathrm {d}}+\Delta D \right) ^2/4$ and $A_{\mathrm {d}}=\pi D_{\mathrm {d}}^2/4$, respectively. Simulating $k_1 \downarrow , k_2 \downarrow$ caused a left-upward shift of the group-averaged *D*-*P* curve, as well as a 40% increase in $C_A$ (Fig. 7). In Fig. 6, distributions of $L_{\mathrm {coll}}$ for the $k_1 \downarrow , k_2 \downarrow$ realisations (red dashed curves) are shown. We found $L_{\mathrm {coll}}$ to equal 0.8 [0.3 ; 2.5]% at $P_{\mathrm {d}}$, 1.7 [0.6 ; 5.0]% at $P_\mathrm {m}$, and 10.8 [7.2 ; 17.6]% at $P_\mathrm {s}$ (median $[{\mathrm {PCI}}_{25\rightarrow 75}]$). As compared to the INIT realisations, spread in $L_{\mathrm {coll}}$ was lower for $k_1 \downarrow , k_2 \downarrow$ realisations (Fig. 6).

## Discussion

Computational models of arterial wall mechanics could be valuable for predicting effects of vascular drug therapies on individual arterial wall constituents. The aim of this study was (1) to quantify how measurement noise propagates into uncertainty of the model predictions and (2) to pinpoint the measurements responsible for the largest spread in mechanical characteristics. The relevance of the model output uncertainty was assessed by simulating the effects of vascular drug treatment on constituent load bearing. To our knowledge, this is the first study to perform rigorous uncertainty quantification and sensitivity analysis, assessing the influence of measurement noise in clinical arterial pressure and diameter measurements on constitutive model predictions.

The present study demonstrates that the clinically usefulness of estimating mechanical characteristics of the carotid artery using a constitutive model is hampered by measurement uncertainty. Using sensitivity analysis, we pinpointed that the majority of uncertainty in mechanical characteristics is caused by uncertainty in measurements of distension and IMT.

**Table 5:** Effect of increasing the number of repeated clinical measurements ($N_\mathrm {rep}$) on collagen load bearing parameters ($L_{\mathrm {coll}}$) as predicted using the initial constitutive model realisations

$N_\mathrm {rep}$	$L_{\mathrm {coll}}$ at $P_{\mathrm {d}}$		$L_{\mathrm {coll}}$ at $P_\mathrm {m}$		$L_{\mathrm {coll}}$ at $P_\mathrm {s}$	
	Median [%]	${\mathrm {PCI}}_{25\rightarrow 75}$ [%]	Median [%]	${\mathrm {PCI}}_{25\rightarrow 75}$ [%]	Median [%]	${\mathrm {PCI}}_{25\rightarrow 75}$ [%]
3	1.5	[0.6 ; 4.5]	3.1	[1.2 ; 8.2]	16.7	[12.7 ; 24.0]
5	1.5	[0.7 ; 3.5]	3.0	[1.4 ; 6.7]	16.5	[13.2 ; 21.8]
10	1.5	[0.9 ; 2.7]	3.0	[1.8 ; 5.3]	16.5	[14.0 ; 19.9]

${\mathrm {PCI}}_{25\rightarrow 75}$: 25th to 75th percentile confidence interval, $P_{\mathrm {d}}$: diastolic blood pressure, $P_\mathrm {m}$: mean blood pressure, and $P_\mathrm {s}$: systolic blood pressure

### Model fitting and parameter estimation

Model fitting was performed on single-exponential diameter–pressure curves calculated from diastolic and systolic diameters and pressures. The assumption of a markedly exponential diameter–pressure curve in the physiological (i.e. diastolic to systolic) pressure range was reported in earlier works by Hayashi et al. (1980) and Meinders and Hoeks (2004). At a wider pressure range (i.e. 40–160 mmHg), a representative constitutive model realisation suggested sigmoidal diameter–pressure behaviour (Fig. 4). Such sigmoidal behaviour was also observed in in vitro studies, performing inflation tests on human aortic segments and rat carotid arteries (Fridez et al. 2003; Langewouters et al. 1986).

In the present study, best-fit constitutive parameter values for elastin stiffness (i.e. 47.4 [44.8 ; 50.0] kPa) were in agreement to those found for cadaveric carotid arteries, reporting values between 20 and 60 kPa (Sáez et al. 2014; Sommer and Holzapfel 2012). Moreover, values for collagen parameter $k_2$ were well within ranges found in earlier studies (Sáez et al. 2014; Sommer and Holzapfel 2012). We found a large spread for constitutive parameter $k_1$, governing collagen stiffness, i.e. median [${\mathrm {PCI}}_{25\rightarrow 75}$] of 2.1 [0.8 ; 5.9] kPa. Previous in vitro studies found $k_1$ values ranging from 2.9 to 99.9 kPa, respectively (Sommer and Holzapfel 2012). Therefore, our findings for $k_1$ are on the low end to those found in the aforementioned studies. It has been pointed out that appropriate choices of both $k_1$ and $k_2$ ensure collagen to virtually not bear load at very low amounts of stretch (i.e. at sub-physiological pressure loads), whereas it will become the dominant load bearer at high amounts of stretch (Holzapfel et al. 2000). In the present study, and in *in vivo* studies *per se*, diameter and pressure measurements at these very low pressures are unavailable, making robust estimation of model parameters (particularly $k_1$) cumbersome, as illustrated by our findings.

### Sensitivity analysis

Sensitivity analysis indicated that the most important contributors to uncertainty in $c_{\mathrm {elast}}$ are both the variables measured by ultrasound (i.e. distension and IMT), whereas uncertainty in collagen parameter $k_1$ was primarily caused by measurement uncertainty of distension. Although our model-based approach still requires blood pressure to be measured, improving the precision of distension and wall thickness measurements clearly appears to be most rewarding. Recent technological advances in vascular imaging, including plane wave ultrasound and image-reconstruction algorithms, could reduce the measurement noise of a single ultrasound measurement (Besson et al. 2016). More practically, uncertainty in mechanical characteristics could be reduced by increasing the number of repeated measurements ($N_\mathrm {rep}$, Eq. 8).

### Decreasing measurement uncertainty by increasing the number of repeated measurements

We evaluated to which extent increasing $N_\mathrm {rep}$, for all measured variables displayed in Table 1, influenced uncertainty in collagen load bearing parameters. Results indicate that increasing the number of repeated measurements from 3 to 10 decreases the spread in $L_{\mathrm {coll}}$ by $\sim 50\%$ (i.e. reducing the 25th to 75th percentile confidence interval at systolic blood pressure from [12.7 ; 24.0]% to [14.0 ; 19.9]%, Table 5). Based on the results of our sensitivity analysis, increasing the number repetitions of ultrasound measurements appears most rewarding in reducing uncertainty in collagen load bearing parameters.

### Model-based assessment of vascular drug therapies

AGE-breaking vascular drug therapy was simulated by changing constitutive model parameters governing collagen behaviour (i.e. parameters $k_1$ and $k_2$). We chose to reduce collagen stress scaling parameter $k_1$, as well as collagen stress curve shape parameter $k_2$ by 40% of their initial best-fit values. Consequently, the modelled collagen becomes incrementally less stiff at low amounts of strain, but will also stiffen “later” (i.e. at higher amounts of strain), compared to when the initial parameters would be used (Fig. 6). This shift in stress–strain behaviour with decreasing cross-link densities was measured also in collagenous tissue such as the pericardium (Kayed et al. 2015; Fratzl 2008). Reducing constitutive parameters $k_1$ and $k_2$ by 40% resulted in a model-predicted area compliance increase from 0.15 $\mathrm {mm}^2/\mathrm {mmHg}$ to 0.21 $\mathrm {mm}^2/\mathrm {mmHg}$ (Fig. 7). This observed 40% increase in area compliance is in agreement with in vitro measurements performed in rat carotid arteries, following AGE-breaker treatment (Wolffenbuttel et al. 1998). Furthermore, collagen load bearing is reduced, with the load transferred to elastin instead (Figs. 6 and 7). The median reduction of collagen load bearing was 1%, 2%, and 6% at diastolic, mean, and systolic blood pressures (Fig. 6). However, the reduction in collagen load bearing was exceeded by the initial spread in collagen load bearing. This is illustrated by the reduction in collagen load bearing at systolic blood pressure (6%, respectively, Fig. 6), which is much smaller compared to the 25th to 75th percentile confidence interval ranging between 12.7 and 24.0%, respectively (Fig. 6). In other words, measurement of the benefit of a cross-link breaker on arterial compliance may be easily concealed by uncertainties in the estimation of the effects, given the impact of noise on model output.

### Limitations

Unfortunately, no actual distensibility measurements, acquired before and during AGE-breaker treatment, were available in this study. Consequently, we had to resort to simulating AGE-breaker treatment using our constitutive model. This was achieved by changing constitutive parameters governing collagen behaviour, reproducing results from an earlier AGE-breaker intervention study (Wolffenbuttel et al. 1998). The Holzapfel-Gasser-Ogden model we used-neglecting active smooth muscle response and containing only three parameters to characterise elastin and collagen behaviour-was chosen as a pragmatic simplification of the actual arterial biomechanical behaviour. Furthermore, our model neglects the dispersion of collagen fibre orientation in the adventitia (Gasser et al. 2006). We are aware that more elaborate models exist describing the influence of collagen cross-links on the stress–strain behaviour of an artery more directly. For example, in the work of Sáez et al. (2014), a cross-linking degree parameter was introduced which includes cross-links behaviour between the main collagen fibres. However, using such a model requires estimating one extra model parameter. To ensure unique parameter values, this would require more clinical data to be measured, which might not be possible in *in vivo* situations. Of note, parameter values in the current study were highly similar, indicated by the fact that the best-fit constitutive parameter values—for each of the 10 random starting points—were highly similar.

In our model, not all combinations of $c_{\mathrm {elast}}$, $k_1$, and $k_2$ yield physiological behaviour. However, the adjustment (fitting) of these parameters ensures that eventually their combination does yield physiological behaviour. The parameter $c_{\mathrm {elast}}$ can be physiologically interpreted as the stiffness of the arterial elastin. All fitted values for this parameter ranged from 35 to 55 kPa, which corresponds to previous literature (Sommer and Holzapfel 2012). For $k_1$ and $k_2$, no separate interpretation can be made in terms of physiology. Nevertheless, the fitted combinations of $k_1$ and $k_2$ yielded physiologically realistic mechanical behaviour which, given that the elastin model was plausibly parameterised, corresponds to realistic collagen behaviour.

The global variance-based sensitivity analysis method used distinguishes from more commonly used local methods by taking into account the entire distribution of measured variables, being model free, and assessing interaction between measured variables (Borgonovo and Plischke 2016; Quicken et al. 2016; Sudret 2015). However, it assumes on the one hand statistical independence between measured variables and on the other hand that variance is an adequate metric for model uncertainty. The latter assumption becomes questionable for skewed distributions of parameters, i.e. as present in the distributions of parameters $k_1$ and $L_{\mathrm {coll}}$ at $P_{\mathrm {d}}$ and $P_\mathrm {m}$, respectively (Figs. 5 and 7). A solution to this problem could be to use moment-independent sensitivity methods instead (Borgonovo and Plischke 2016). To this end, Borgonovo (2007) evaluated an alternative sensitivity metric that, instead of being computed using the variance of the model uncertainty distribution, considers this distribution as a whole. In their paper, it was concluded that sensitivity indices of both methods (1) show discrepancies between influential measured variables, but (2) agree in distinguishing non-influential from influential measured variables (Borgonovo 2007). Based on these findings, we believe that utilising the variance-based sensitivity analysis as proposed by Quicken et al. (2016) in this study is justified.

### Conclusion

This study shows that *in vivo* assessment of arterial wall mechanics using a constitutive model is hampered by large model uncertainty. We quantified model uncertainty in constitutive parameters (i.e. $c_{\mathrm {elast}}$, $k_1$, and $k_2$, respectively), and collagen load bearing parameters, at various blood pressures $(L_{\mathrm {coll}}$). Our simulation of vascular drug therapy suggested a reduction of collagen load bearing of 6-percentage points, at systolic blood pressure. This reduction is 3–4 times lower compared to its uncertainty. Therefore, model output uncertainty could conceal potential effects of vascular drugs. Sensitivity analysis revealed that estimation of mechanical characteristics would benefit most from increasing the precision of measurements of arterial diameter, distension, and wall thickness. Whereas the potential for improving the precision of, for example, a single ultrasound measure is practically limited, the effective precision of ultrasound measurements could be improved by increasing the number of repeated measurements.

## Appendix: Constitutive model

### Constitutive relations

Under the assumptions that (1) elastin and collagen act in parallel in terms of mechanical load bearing, (2) the vessel wall is incompresible, and (3) deformation occurs only along the principal axes, the constituent-specific strain energy functions (Eqs. 1 and 2) were cast into a relation describing local Cauchy stress:

A1 $$\begin{aligned} \sigma _n = -P_{\mathrm {h}} + \lambda _n \frac{\partial W_{{\mathrm {elast}}}}{\partial \lambda _n}+\lambda _n \frac{\partial W_{{\mathrm {coll}}}}{\partial \lambda _n} ~{\mathrm {,}} \end{aligned}$$

The subscript *n* is *rr* for the radial direction, $\theta \theta$ for the circumferential direction and *zz* for the axial direction. This results in the following expressions for $\sigma _{rr}$, $\sigma _{\theta \theta }$ and $\sigma _{zz}$:

A2 $$\begin{aligned}&\sigma _{rr} = -P_{\mathrm {h}} + 2\lambda ^2_{rr} c_{\mathrm {elast}}~{\mathrm {,}}\nonumber \\&\sigma _{\theta \theta } = -P_{\mathrm {h}} + 2\lambda ^2_{\theta \theta } c_{\mathrm {elast}} \nonumber \\&\quad +\,4k_1\lambda ^2_{\theta \theta }(\lambda ^2_{\mathrm {fibre}}-1)\cos ^2(\beta _0) \left( \exp \left[ k_2\left( \lambda ^2_{\mathrm {fibre}}-1\right) ^2 \right] \right) ,\nonumber \\&\mathrm {and}\nonumber \\&\sigma _{zz} = -P_{\mathrm {h}} + 2\lambda ^2_{zz} c_{\mathrm {elast}}\nonumber \\&\quad +\,4k_1\lambda ^2_{zz}(\lambda ^2_{\mathrm {fibre}}-1)\sin ^2(\beta _0) \left( \exp \left[ k_2\left( \lambda ^2_{\mathrm {fibre}} - 1\right) ^2\right] \right) \nonumber \\ \end{aligned}$$

with $P_\mathrm {h}$ the local hydrostatic pressure within the wall, $\lambda _{\mathrm {fibre}}$ the fibre stretch given by $\lambda _{\mathrm {fibre}} = \sqrt{\cos ^2 (\beta _0) \lambda ^2_{\theta \theta } + \sin ^2 (\beta _0) \lambda ^2_{zz}}$, $\beta _{0}$ the fibre helix angle, $c_{\mathrm {elast}}$ the constitutive (stiffness) parameter of elastin, and $k_1$ and $k_2$ stiffness parameters of collagen, respectively.

### Constitutive framework

We distinguished between the unstressed and stressed states of an artery (Fig. 2). Under the assumption of an incompressible wall tissue, a mapping of coordinates $(r,\theta , z)$ of the deformed, stressed configuration to coordinates $(R,\Theta , Z)$ of the unstressed configuration is given by:

A3 $$\begin{aligned} r(R) = \sqrt{r^2_{\mathrm {o}} - \frac{R^2_{\mathrm {o}} - R^2}{\lambda _{zz} k_\alpha }} ~{\mathrm {,}}~ \theta =k_\alpha \Theta ~\mathrm {,~and}~ z = \frac{l}{L}Z ~{\mathrm {,}} \end{aligned}$$

where $R_{\mathrm {o}}$ and $r_{\mathrm {o}}$ are the outer radii of respectively the unstressed and stressed configuration (Fig. 2). The parameter $k_\alpha$ is defined as $k_\alpha = \frac{2\pi }{2\pi - \alpha }$, with $\alpha$ the opening angle (Fig. 2). *L* and *l* are vessel length of the unstressed and stressed configuration defining the initial pre-stretch of an artery $\lambda _{zz}$ (Fig. 2). The principal stretch ratios ($\lambda _{rr},\lambda _{\theta \theta },\lambda _{zz}$) with respect to the unstressed configuration are:

A4 $$\begin{aligned} \lambda _{rr} = \frac{R}{r k_\alpha \lambda _{zz}}, \quad \lambda _{\theta \theta } = \frac{k_\alpha r}{R}, \quad \mathrm {and} \quad \lambda _{zz} = \frac{l}{L} ~{\mathrm {.}} \end{aligned}$$

Inner and outer radii ($r_{{\mathrm {i}}}$ and $r_{\mathrm {o}}$, respectively) in the stressed configuration can be written as follows:

A5 $$\begin{aligned} r_{{\mathrm {i}}} = \frac{\lambda _{\theta \theta } R_{{\mathrm {i}}}}{k_\alpha } \quad \mathrm {and} \quad r_{\mathrm {o}} = \sqrt{\frac{A_{\mathrm {w}}}{\pi } + r^2_{{\mathrm {i}}}} {{\mathrm {,}}} \end{aligned}$$

where $A_{\mathrm {w}}$ is the cross-sectional wall area in the stressed configuration (Fig. 2). In practice, $A_{\mathrm {w}}$ can be determined by measuring IMT and vessel diameter using ultrasonography (Hoeks et al. 1999). We furthermore assumed $\lambda _{zz} = 1.20$, $\alpha = 100^\circ$, and $\beta _0 = 35.3^\circ$.

### Balance equations

Conservation of momentum is given by

A6 $$\begin{aligned} \nabla \cdot \varvec{\sigma } = \mathbf {0} ~{\mathrm {,}} \end{aligned}$$

with $\nabla$ the spatial gradient operator. Assuming axisymmetry and neglecting torsion, acceleration forces, body forces and axial extension, Eq. A6 reduces to

A7 $$\begin{aligned} \frac{\partial \sigma _{rr}}{\partial r} + \frac{\sigma _{rr} - \sigma _{\theta \theta }}{r} = 0 ~{\mathrm {.}} \end{aligned}$$

Applying the boundary conditions $\sigma _{rr}(r_{{\mathrm {i}}}) = -P$ and $\sigma _{rr}(r_{\mathrm {o}}) = 0$, the expression for lumen pressure (*P*) is given by

A8 $$\begin{aligned} P = \int _{r_i}^{r_o} \frac{\sigma _{\theta \theta }-\sigma _{rr}}{r} {\mathrm {d}}r ~{\mathrm {.}} \end{aligned}$$

Reduced axial force is given by

A9 $$\begin{aligned} F_z =\pi \int _{r_i}^{r_o} (2\sigma _{zz} - \sigma _{rr} - \sigma _{\theta \theta })r {\mathrm {d}}r ~{\mathrm {.}} \end{aligned}$$

### Calculating collagen load bearing parameters

We calculated collagen load bearing ($L_{\mathrm {coll}}$) at three blood pressure levels: diastolic, mean and systolic blood pressure (i.e. $P_{\mathrm {d}}$, $P_\mathrm {m}$, and $P_\mathrm {s}$, respectively). Mean blood pressure ($P_\mathrm {m}$) was calculated as $\frac{1}{3}P_\mathrm {s} + \frac{2}{3}P_{\mathrm {d}}$. Using Eq. A2, the expression for $L_{\mathrm {coll}}$ is given by

A10 $$\begin{aligned} L_{\mathrm {coll}} = \int _{r_{{\mathrm {i}}}}^{r_{\mathrm {o}}} \frac{\tau _{\theta \theta ,{\mathrm {coll}}}}{\sigma _{\theta \theta }-\sigma _{rr}} {\mathrm {d}}r \bigg |_{P \in \{P_{\mathrm {d}},P_\mathrm {m},P_\mathrm {s}\}} \cdot 100\%~{\mathrm {,}} \end{aligned}$$

with $\tau _{\theta \theta ,{\mathrm {coll}}}$ the circumferential stress borne by collagen, given by

A11 $$\begin{aligned} \tau _{\theta \theta ,{\mathrm {coll}}} = \lambda _{\theta \theta } \frac{\partial W_{\mathrm {coll}}}{\partial \lambda _{\theta \theta }} ~{\mathrm {.}} \end{aligned}$$
